# Spinal Subdural Abscess Mimicking an Extradural Tumor: A Case Report

**DOI:** 10.7759/cureus.19101

**Published:** 2021-10-28

**Authors:** Nikolay A Konovalov, Stanislav Kaprovoy, Dmitry Asyutin, Petr Zelenkov, Evgeny Brinyuk

**Affiliations:** 1 Department of Spinal and Peripheral Nerve Surgery, Burdenko Neurosurgical Center, Moscow, RUS

**Keywords:** s. aureus, spinal subdural abscess, extradural tumor, spinal abscess surgery

## Abstract

Spinal subdural abscesses (SSAs) are rare pathologies presenting as encapsulated pus located intradurally and extramedullary. Although there is no uniform opinion on the cause of this pathology, approximately 50% of cases are attributed to *Staphylococcus aureus* infection. Here, we present a rare case of a female patient who presented to N.N. Burdenko Neurosurgical Center for treatment of an extradural tumor in the lower lumbar spine. She complained of acute lower back pain, lower limb muscle spasms, progressive lower limb weakness, numbness in toes, and increased frequency of defecation (five to six times per day). Intraoperatively, we discovered that the epidural space was clear and a subdural abscess was located and removed. The patient was started on antibiotics and recovered 29 days later. This case report illustrates an atypical SSA mimicking an extramedullary tumor on MRI.

## Introduction

Spinal subdural abscesses (SSAs) are considered an extremely rare pathology in spinal surgery. Mostly attributed to *Staphylococcus aureus* infection, they present as demarcated collections of pus located intradurally and most commonly in the lumbar spine [1-3]. Patients often present with fever, back pain, and symptoms of spinal cord compression, which resolve once the SSA has been addressed. The most typical cause is *S. aureus* (50% of cases), although other pathogenic organisms can also cause SSAs [2-4].

Although MRI is the study of choice, imaging characteristics of SSAs on MRI are not well described. Basic findings include a space-occupying, variably enhancing mass in the spinal canal. Fat-suppression and diffusion-weighted images (DWI) aid in diagnosing SSAs [2,5].

The treatment of choice for SSAs is decompressive surgery (laminectomy) with antiseptic irrigation and drainage of the subdural space followed by appropriate antibiotic therapy. Cultures should be obtained before wound irrigation for the definitive diagnosis of the pathogenic organism [2,3,6,7]. Here, we describe a case of SSA caused by *S. aureus* that was treated at our facility.

## Case presentation

A 49-year-old female was referred to our department for the surgical treatment of an extradural tumor, with complaints of acute lower back pain, lower limb muscle spasms, progressive lower limb weakness, numbness in toes, and increased frequency of defecation (five to six times per day). Symptoms manifested approximately 1.5 months prior to admission with an acute onset of lower back pain, for which she was treated conservatively with no clinical effect. An MRI scan of the lumbar spine revealed an extensive extradural tumor at the level of Th12-S2 vertebrae, covering the dural sack and roots with a right-sided presacral paravertebral spread at the S2-S3 level. She had no surgical history and was hospitalized with right lower limb venous thrombosis, mild urinary infection, and chronic colitis, which were not considered a surgical risk.

**Figure 1:**
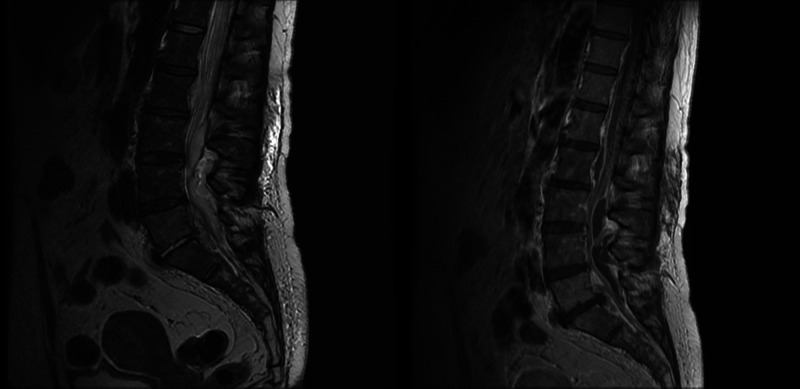
Sagittal T1 and T2-weighted MRI showing an extradural tumor along with the presence of a soft tissue, heterogeneously contrast-enhancing mass, enveloping the roots of the cauda equina, located predominantly at the Th12-L2 level along the anterior and the L3-L5 level on the posterior surface of the dural sac. MRI: magnetic resonance imaging

Upon admission, secondary MRI scans revealed an extradural mass-like lesion at the level of Th12-L5 vertebrae and another lesion at the S2-S3 level (Figure 1). Considering the patient’s history and MRI findings, we suspected that the lesion might be a malignant peripheral nerve sheath tumor at the S2-S3 level with secondary metastases into the spinal canal. Neurologic examination revealed lower limb paraparesis (3 points on the Medical Research Council [MRC] score), bilateral decreased reflexes, bowel dysfunction with imperative urges, and bilateral positive Babinski sign. The patient had no fever, chills, or meningeal irritation. No paraspinal muscle tenderness was detected on percussion.

On laboratory tests, inflammatory markers were elevated (erythrocyte sedimentation rate [ESR]: 21 mm/hour; normal: 2-15 mm/hour; C-reactive protein [CRP]: 31.1 mg/L; normal: 0.1-5.0 mg/L; white blood cell [WBC] count 10.80 ×10^9^/L; normal: 3.40-10.80 ×10^9^/L). This was initially attributed to the patient’s urinary tract infection (UTI). Urinalysis on admission showed an increased WBC count (152.80 cells/µL; normal: 0.00-39.00 cells/µL) and bacteria in urine sample (2,845.60 cells/µL; normal: 0.00-386.00 cells/µL). The patient was started on factor Xa inhibitors for thrombosis, stopped 12 hours prior to surgery, peroral baclofen for lower limb muscle spasms, and peroral norfloxacin for her UTI. Urinalysis performed on the morning before surgery showed a marked decrease in WBC count (77.20 cells/µL; normal: 0.00-39.00 cells/µL) and no bacteria in the urine sample.

**Figure 2:**
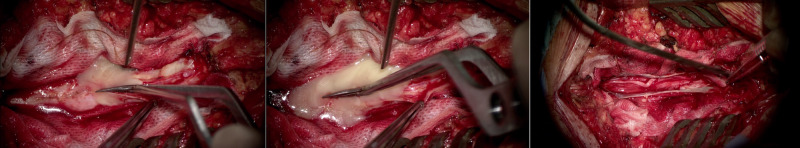
Intraoperative image showing whitish cheese-like pus pouring out into the epidural space.

Two days after admission, an L3-S1 laminectomy was performed. Upon inspection, the epidural space was clear and a tensed dura was noted. After durotomy, whitish cheese-like pus poured out into the epidural space. The abscess capsule was found to be closely adherent to the epidural space (Figure 2).

**Figure 3:**
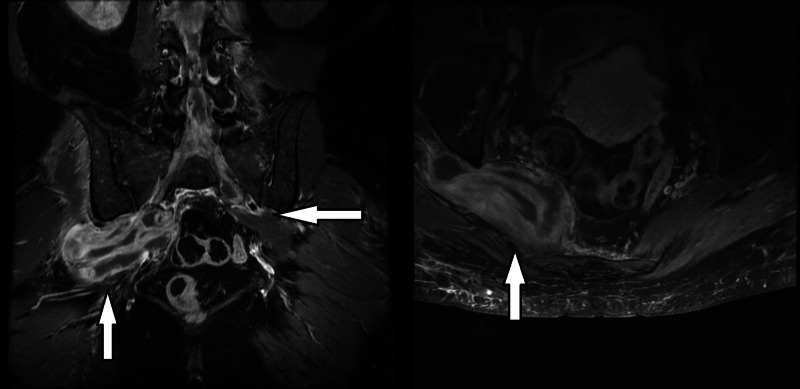
Sagittal and axial T1-weighted MRI obtained preoperatively showing a large intrapelvic abscess on the right side and a small abscess on the left side. MRI: magnetic resonance imaging

The abscess was removed and extensive irrigation with an antiseptic solution was performed. An express microbiological examination of the pus revealed *S. aureus*. After abscess removal, the dura was closed and an external flow-through wound drain was inserted. The wound was irrigated with 1.2 L of antiseptic solution (diluted iodine solution) daily for 15 days. Intravenous (IV) vancomycin 1 g every six hours was administered for 12 days, followed by IV amoxicillin 1.2 g every eight hours for 14 days based on antibioticogram results. After the surgery, the preoperative MRI was thoroughly analyzed. One large and one small abscess in the pelvis were found, which we missed preoperatively and considered being an extension of the spinal abscess into the pelvis (Figure 3).

**Figure 4:**
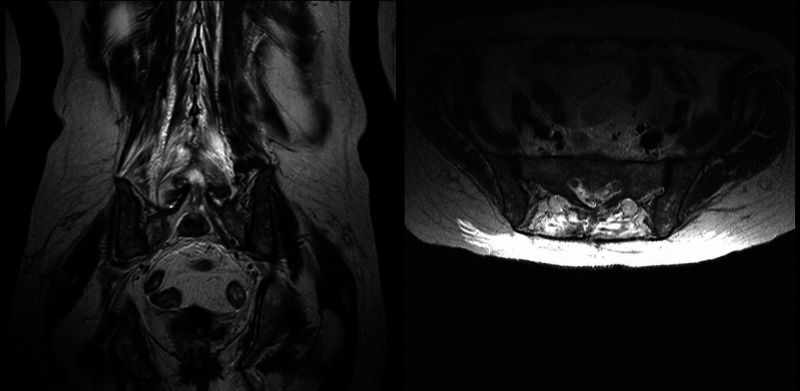
Sagittal and axial T1-weighted MRI obtained upon discharge showing no signs of intrapelvic abscesses on either side. MRI: magnetic resonance imaging

Postoperatively, the patient’s symptoms improved. At discharge, the patient suffered no lower back pain or muscle spasms, her lower limb strength increased (4 points on MRC score), and bowel dysfunction regressed completely. The patient was discharged 22 days after the surgery, with two consecutive microbiological examinations of wound content taken from the drainage system verified as sterile. Upon discharge, a control MRI scan was obtained. No pelvic abscess was found on the MRI scan (Figure 4). Treatment with amoxicillin was continued seven days after the dischage at the same regimen.

## Discussion

SSA is a rare and unpredictable type of infectious intradural extramedullary lesion (IDEM). To our knowledge, its exact incidence is unknown [4,7]. It is considered a neurosurgical emergency and is associated with high morbidity and mortality rates [5]. The current understanding of the pathophysiology, diagnosis, and treatment of SSAs is based on anecdotal evidence due to the rarity of the condition [2]. Since the first description by Sittig in 1927, 138 cases have been published in the literature [2,8].

The lumbar spine is the most common region for SSAs which are typically attributed to *S. aureus* (over 50% of reported cases). Other pathogenic organisms include various *Staphylococcus* species, *Streptococcus*, *Escherichia coli*, *Pseudomonas aeruginosa*, *S. pneumoniae*, and *Peptococcus magnus* [1-3,5]. In our case, the pathogenic organism responsible was a wild-type *S. aureus* that caused the SSA that spread into the pelvis. To our knowledge, there are no reported cases of spinal intradural abscesses associated with abscesses in the pelvic region.

Clinical signs and laboratory findings of spinal infections can be subtle and misleading [5]. Fraser et al. described the classical triad of symptoms of an SSA in 1973, which included fever, neck/back pain, and symptoms of spinal cord/cauda equina compression, namely, pain, numbness, and/or muscle weakness in one or both legs, bladder/bowel dysfunction, and erectile dysfunction [2,4]. Bartels et al. in their review of 44 SSA cases found that, at initial symptom onset, 84.4% of patients presented with spinal/limb pain and 55.6% with fever. By the time patients presented for medical care, 86.7% had fever, 84.4% had spinal/limb pain, and 82.2% had a motor deficit [2,6].

A key diagnostic feature of SSA is the absence of spinal muscle tenderness on spinal percussion, which helps differentiate this process from the more common spinal epidural abscess; however, Levy et al. found 14 of the 47 patients they reviewed to have spinal tenderness [2,9]. In our case, the patient presented with acute lower back pain, mild motor and sensory deficits, and bowel dysfunction. She had no fever, and no spinal muscle tenderness was found on percussion. This may be attributed to the second stage of the disease process reported by Bartels et al. [2,6].

Leukocyte count, ESR, and CRP are not sensitive indicators of spinal infection but are usually elevated [3]. In our case, leukocyte count was normal and ESR and CRP were elevated, which was attributed to concomitant urinary infection.

The definitive diagnostic study of choice is a contrast-enhanced MRI. It allows good visualization of the spinal cord, bony and ligamentous tissues, intervertebral discs, the extent of lesion, and compression. Because there are only a few published cases on imaging characteristics of SSAs, exact MRI findings are not well described. Although MRI is the study of choice, SSA is a surgical diagnosis as it may be difficult to differentiate subdural from epidural infection. Fat-suppression sequence and DWI can be useful tools in differentiating abscesses from tumors [2,3,5].

In accordance with the aphorism “ubi pus, ibi evacua,” surgery followed by appropriate antibiotic therapy is considered the mainstay of treatment. Prompt decompression (laminectomy) with antiseptic irrigation and drainage of the subdural space, followed by antibiotic therapy is considered the conventional treatment approach. Pus cultures for identifying a definitive pathogenic organism are mandatory and should be obtained before wound irrigation. Most studies advocate primary water-tight dural closure. In regards to placing external drains for postoperative irrigation, this has not been adopted as a part of the conventional treatment approach. Although there is some data concerning successful conservative treatment, overall prognosis is considerably better for patients who undergo surgical treatment. Bartels et al. reviewed 45 patient treated for SSAs. In the surgical group, 82.1% completely recovered or improved and 17.9% died. In the conservative group, 80% died and 20% improved. These numbers correlate with the current treatment mainstay for aggressive surgical treatment followed by antibiotic therapy [2-4,6,7].

## Conclusions

In our case, the patient had no obvious evidence of SSA and was misdiagnosed with an IDEM tumor. We consider this to be caused by an atypical presentation of SSA associated with an abscess in the pelvic region. A thorough inspection is needed in the presence of heterogeneous diffuse lesions on MRI in the lumbar region.
